# Spatial transcriptomic sequencing reveals immune microenvironment features of *Mycobacterium tuberculosis* granulomas in lung and omentum

**DOI:** 10.7150/thno.99038

**Published:** 2024-09-23

**Authors:** Xiaochen Qiu, Pengfei Zhong, Liang Yue, Chaofan Li, Zhimin Yun, Guangqian Si, Mengfan Li, Zhi Chen, Yingxia Tan, Pengtao Bao

**Affiliations:** 1 The Eighth Medical Center, Chinese PLA General Hospital, 100039, Beijing, China.; 2 Senior Department of General Surgery, Chinese PLA General Hospital, Beijing, 100093, China.; 3 Graduate School, Hebei North University, 075000, Zhangjiakou, Hebei Province, China.; 4 Academy of Military Medical Sciences, Beijing, 100850, China.; 5 Senior Department of Tuberculosis, Chinese PLA General Hospital, Beijing, 100093, China.; 6 Senior Department of Pulmonary and Critical Care Medicine, Chinese PLA General Hospital, Beijing, 100093, China.

**Keywords:** Granulomas, Spatial transcriptomics techniques, Pulmonary tuberculosis, Omental tuberculosis, Lung organoid-macrophage co-culture model, *In vitro* tuberculosis model

## Abstract

Granulomas are a key pathological feature of tuberculosis (TB), characterized by cell heterogeneity, spatial composition, and cellular interactions, which play crucial roles in granuloma progression and host prognosis. This study aims to analyze the transcriptome profiles of cell populations based on their spatial location and to understand the core transcriptome characteristics of granuloma formation and development.

**Methods** In this study, we collected four clinical biopsy samples including *Mycobacterium tuberculosis* (Mtb) infected lung (MTB-L) and omentum tissues (MTB-O), as well as two lung and omentum biopsies from non-TB patients. The tissues were analyzed by spatial transcriptomics to create a spatial atlas. Utilizing cell enrichment scores and intercellular communication analysis, we investigated the transcriptome signatures of cell populations in various spatial regions and identified genes that may play a decisive role in the formation of pulmonary and omental tuberculosis granulomas. To validate our major findings, an *in vitro* TB model based on organoid-macrophage co-culture was established.

**Results** Spatial transcriptomics mapped the cell composition and spatial distribution characteristics of tuberculosis granulomas in lung and omental tissues infected with Mtb. The characteristics and evolutionary relationships of major cell populations in granulomas reveal a shift in the immune microenvironment: from a predominance of B cells and fibroblasts in pulmonary granulomas to a predominance of myeloid cells and fibroblasts in omental granulomas. Furthermore, our data identified key differentially expressed genes across cell clusters and regions, showing that upregulation of collagen genes is a common feature of granulomas. Using an organoid-macrophage co-culture model, we demonstrated the notable efficacy of Thrombospondin-1 (THBS1) in reducing protein expression levels related to extracellular matrix remodeling.

**Conclusion** These results provide insights into the pathogenesis and development of tuberculosis, enhancing our understanding of the composition and interactions of tuberculosis granuloma cells from a spatial perspective, and pave the way for novel adjuvant treatments for tuberculosis.

## Introduction

Tuberculosis (TB) remains one of the most common causes of death from a single infectious disease. An estimated 10.0 million people were found to develop TB globally, with 1.2 million TB-associated deaths among HIV-negative people and an additional 208, 000 deaths among people living with HIV in 2019 1. Granulomas represent the pivotal pathological structure in tuberculosis, exhibiting significant heterogeneity in cellular composition and spatial distribution. These patterns reflect the immune status of affected organs 2. Macrophages, T cells, B cells, fibroblasts, and extracellular matrix components collectively form the granuloma framework. The mechanism underlying granuloma formation, driven by immune cells and fibroblasts, has been extensively explored. The intricate interplay among these cells maintains the inflammatory milieu and structural integrity of granulomas. Conventionally, granuloma formation was viewed as confining bacteria to specific sites and controlling their proliferation. Emerging evidence suggests that the highly encapsulated structure of mature granulomas may impede the infiltration of immune cells and antituberculosis drugs, allowing bacteria to persist in a dormant state, awaiting opportunities for reactivation 3. Notably, the progression of highly fibrotic granulomas can lead to tissue remodeling, compromising organ function 4. Consequently, there is growing recognition that a subset of granuloma outcomes may serve as the starting point for tuberculosis and significantly influence its progression. However, the detrimental factors driving granuloma progression remain incompletely understood. Given the close relationship between inflammatory signaling and spatial organization within granulomas, observing molecular events from a spatial perspective is essential for better discerning and interpreting the similarities and differences in cellular composition and interactions within granulomas across various tissues. With ongoing advancements in molecular biology technology, higher resolution dimensional sequencing, and fluorescence imaging offer opportunities for obtaining more detailed insights into granuloma biology.

In the current study, we generated a full spatial gene expression atlas by employing spatial transcriptomics (ST) analysis to decipher the potential mechanisms underlying the formation of TB lesions in different tissues. ST enables the quantification of the mRNA population within intact tissue, offering unbiased mapping of transcripts over the entire tissue and providing novel insights into the molecular processes in diseases 5. Our samples were taken from lung tissue and omentum, representing different organ and immune features, respectively. The pulmonary sample represents the primary tuberculosis lesion, while Peritoneal TB is thought to develop from the re-activation of latent foci of infection established in the peritoneum, and spread hematogenously to the mesenteric lymph node from a previous pulmonary infection 6. Our findings unveiled significant heterogeneity within cell populations between pulmonary and omental granulomas and these findings would provide a foundation for further exploration of tuberculosis granulomas, particularly in alleviating tissue injury and decline in organic function caused by severe fibrosis of tuberculosis granulomas.

## Methods

### Clinical samples information

Four clinical samples (slides of tissue blocks) were obtained from the Pathology Department of Chinese PLA General Hospital including one case of pulmonary tuberculosis (MTB-L), one case of omental tuberculosis (MTB-O), one case of lung tissue (CTR-L) and one case of normal omentum tissue (CTR-O) with non-TB diseases as a control. The clinical samples were acquired in accordance with institutional review board protocol (Ethical Number 3092023006290920), and the additional data are provided in Table 1.

### Spatial transcriptomics sequencing and analysis

The tissues were transferred to isopentane for soaking and freezing, and moved to a cryopreservation tube with tweezers for subsequent embedding in paraffin. The procedures are as follows: (1) Hematoxylin-eosin staining (HE) was used for histological imaging; (2) Tissues were then fixed, stained and permeabilized to release mRNA, which can bind to the probes that contained a 16 bp spot barcode and a 12 bp UMI sequence. The capturing probe with poly (dT) sequence acquired gene expression information by binding to mRNA 3'-poly(A) tail; (3) cDNA synthesis and sequencing libraries were prepared using the captured RNA as templates; (4) The sequencing process was based on 10× Genomics Visium and the paired-end sequencing mode of the Illumina sequencing platform.

After sequencing, the data were visualized and analyzed via Space Ranger (version1.1.0). The Space Ranger was used to ensemble the reference genome database and FastQC software was utilized for data quality control. Further sequencing and the application of R software and other applications were used for data visualization. R software version 4.0.3 and “Seurat” software package version 3.1.1 were used for analysis. After excluding low-quality units, we used the “SCTransform” function to normalize data, find variable features and scale data.

### Spatial trajectory inference

stLearn is designed to comprehensively analyze Spatial Transcriptomics (ST) data to investigate complex biological processes within an undissociated tissue. stLearn can exploit all three data types: Spatial distance, tissue Morphology, and gene Expression measurements (SMEs) from ST data. stLearn extracts morphological features from an H&E image that accompanies certain spatial transcriptomics technologies to perform spatial smoothing on the expression data, with which it performs spatial domain detection and further trajectory inference on pairs of spatial domains 7.

### Cell-cell communication analysis

CellChat contains ligand-receptor interaction databases for human and mouse that can analyze the intercellular communication networks from scRNA-seq data annotated as different cell clusters 8. First, we used CellChat to evaluate the major signaling inputs and outputs among all clusters using CellChatDB.human. Then, we used the netVisual_circle function to show the strength or weakness of cell-cell communication networks from the target cell cluster to different cell clusters in all clusters. Finally, the netVisual_bubble function shows the bubble plots of significant ligand-receptor interactions between the cell cluster.

### Bacterial strains and cell culture

*Mycobacterium tuberculosis* (Mtb) *H37Rv* strain (CMCC93009, JZ101702, Hunan Fenghui biological Co.) used in this study was cultured on Middle Brook 7H11 Agar Base (LA7240, Solarbio) supplemented with 10% Middlebrook OADC Growth Supplement (LA9560, Solarbio) and 0.5% Glycerol at 37℃. When the bacteria reached mid-exponential phase, they were harvested in saline, dispersed and quantified by a bacterial ultrasonic dispersion counter device (BACspreader1100, TB Healthcare, China). The Mtb suspension was then adjusted to a cell concentration of 1 MCF. This instrument disperses bulk bacterial clones into single cells by sonication and counts the cells.

The monocyte cell line THP-1 were purchased from ATCC and cultured in RPMI-1640 medium (Gibco) supplemented with 10% FBS, 0.05 mM β-mercaptoethanol, and 1% penicillin/streptomycin (P/S). After seeding for 24 hours, phorbol 12-myristate 13-acetate (PMA, MedChem Express, USA) was added at a concentration of 100 nM for 48 hours to induce differentiation of THP-1 cells from monocytes to macrophages. To collect macrophages, we needed to replace the medium with serum-free medium one day in advance. Then, the cells were washed and digested with trypsin, and the concentration was adjusted before co-culture with the lung organoids.

### Generation of 3D lung organoids from human iPSCs

To generate 3D lung organoids from human induced pluripotent stem cells (iPSCs), we followed the protocol established by Leibel SL. Initially, human iPSCs (RC01001-A, Male, Nuwacell Biotechnologies Co., Ltd, China) were differentiated into lung progenitor cells. When the human iPSCs reached 70% confluence (Day 0), the cells were dissociated into single cells after being digested with accutase (Innovative Cell Technologies) for 20 minutes at 37 °C. 1.75×10^5^ iPSCs were seeded on Matrigel-coated plates in DE induction medium (RPMI1640 (Life Technologies), 1× B27 supplement, 1% HEPES, 1% glutamax and 50 U/mL of penicillin/streptomycin, activin A (100 ng/mL, R&D systems), CHIR99021 (5 μM, Stemgent), and 10 μM of Y-27632). On days 2-4, only activin was added. On day 5, the cells were cultured in anterior foregut endoderm (AFE) medium and induced to differentiate into AFE cells. The basal mediums consisted of 3:1 IMDM:F12 (Life Technologies), 1× B27 and N2 supplements (Life Technologies), 50 U/mL of penicillin/streptomycin, 0.25% BSA, 50 μg/mL of L-ascorbic acid (Sigma-Aldrich), and 0.4 mM of monothioglycerol (Wako). The basal medium was supplemented with SB431542 (10 μM, R&D Systems) and Dorsomorphin (2 μM, Stemgent) for AFE induction for 3 days. The AFE cells were then further differentiated into lung progenitor cells for 10 days in lung progenitor cell (LPC) induction medium containing 10 ng/mL BMP4, 0.1 μM retinoic acid (Sigma-Aldrich), and 3 μM CHIR99021. Afterward, the LPC cells were dissociated, mixed with Matrigel (Corning), and seeded in a 24-well plate. 3D organoid induction medium (the basal medium with FGF7 (10 ng/mL), FGF10 (10 ng/mL), CHIR99021 (3 μM) and EGF (10 ng/mL)) was added for 6 days, the lung organoids formed spheroids with 3D structures. Then the medium was changed to 3D organoid branching medium consisting of FGF7 (10 ng/mL), FGF10 (10 ng/mL), CHIR (3 μM), RA (0.1 μM), EGF (10 ng/mL) and VEGF/PIGF (10 ng/mL) for 6 days, led to the development of bud tip stage organoids with cavities and thin walls. Finally, the organoids were then matured into fully developed lung organoids with the addition of dexamethasone (50 nM), cAMP (100 μM) and IBMX (100 μM).

### Human lung organoid-macrophage co-culture model

To co-culture lung organoids with immune cell-macrophages in Matrigel, the organoids were first gently separated from the Matrigel using a trimmed pipette tip. Subsequently, macrophage suspension (containing 2×10^5^ cells) was added and mixed with lung organoids. The samples were then centrifuged at 1,000 rpm for 3 minutes at 4°C. After removing the medium, the organoids and macrophages were resuspended in 1mL Matrigel on ice. The mixture was promptly reseeded into a 24-well plate (50 µL per well) as soon as possible to prevent the organoids from settling. Finally, the plates were placed in a 37°C, 5% CO_2_ incubator for 30 minutes to allow the Matrigel to solidify. After solidification, organoid medium was added to establish the co-culture.

### Infection of lung organoids with Mtb

To create an *in vitro* model of Mtb infection, lung organoids were transferred to a new 24-well plate and supplemented with 1 mL of organoid culture medium without antibiotics. Subsequently, 1×10^4^ macrophages were added to each well. The culture model was divided into three groups: the Uninfection group (not infected with Mtb), the Mtb infection group (infected with 1×10^7^ CFU/mL Mtb suspension), and the Mtb+LSKL group (pre-treated with 10 ng/mL LSKL inhibitor for 12 hours before adding 1×10^7^ CFU/mL Mtb suspension). Following 24 hours of co-incubation, the cells and organoids were washed with DPBS, centrifuged and resuspended with Matrigel, and plated in a 24-well plate. The plate was then incubated at 37°C for 30 minutes to allow the gel to solidify, followed by an 8-day incubation period in organoid culture medium, with medium changes every 2-3 days. The Mtb+LSKL group received continuous supplementation with 10 ng/mL LSKL inhibitor. After 8 days, the culture model was partially fixed in 4% paraformaldehyde for immunofluorescence analysis and partially stored at -80°C for protein extraction.

### Acid-fast staining

The tissue sections were stained with Ziehl-Neelsen stain (Solarbio) at room temperature for 4 hours after dewaxing. Then, the sections were washed with running water for 5 minutes and decolorized using a differentiation solution containing 1% hydrochloric acid for more than 10 seconds. Hematoxylin was then used to counterstain the nuclei for 5 minutes, after which the excess stain was rinsed off. Dehydration was performed before sealing the sections with neutral resin glue.

### Multiplex fluorescence *in situ* hybridization

In order to assess the levels of CD36, THBS1/2, and CD68 mRNA expression in tuberculosis granuloma lesions, we performed *in situ* fluorescence hybridization on tissue sections from tuberculosis patients. The paraffin-embedded tissue sections were deparaffinized in xylene and ethanol, followed by antigen retrieval using citric acid-EDTA solution. Proteinase K digestion was then carried out, and the sections were incubated in a prehybridization solution before hybridization with probes overnight (Probes information is shown in Table 2). After hybridization, the sections were washed with SSC buffers and subjected to branch probe hybridization. Finally, the sections were stained with DAPI, sealed with anti-fluorescence quencher, and imaged under a fluorescence microscope. The sequences of the fluorescent probes and their corresponding signal channels were utilized for detection.

### Immunofluorescence and immunohistochemical analyses

Lung organoids were fixed in 4% paraformaldehyde, and embedded in paraffin to prepare paraffin sections. For the immunofluorescence assay, sections were blocked using 10% goat serum (Thermo, 31872) prior to primary antibody labeling with antibodies diluted in 10% goat serum, followed by sequential PBS wash, and secondary labeling with AlexaFluor conjugates. Samples were mounted with fluorescence mounting medium (Dako, S3023) and imaged with an inverted or confocal laser-scanning microscope (NIKON A1R).

To verify the protein expression of CD3, CD20, CD36, and THBS1/2 in tuberculosis lesions, paraffin sections were stained using the TSAPlus staining kit (G1255-50T, ServiceBio). After dewaxing and antigen retrieval, the sections were incubated with the primary antibody overnight at 4°C. Following sequential PBS washes, an HRP-labeled secondary antibody was applied. Then the sections were incubated with the iF440-TSA working solution for 10 minutes in the dark. To remove previously bound antibodies, the sections were boiled in citrate buffer for 8-10 minutes and blocked with 3% BSA for 30 minutes before incubating with the second primary antibody overnight at 4°C. The staining process was repeated with iF488-TSA, iF594-TSA, and iF647-TSA working solutions. After adding an anti-fluorescence quencher, the slides were sealed and images were captured using a fluorescence scanner.

The VECTASTAIN® ABC immunohistochemical detection kit (Vectorlabs) was used to perform THBS-1/2 immunohistochemical staining following the manufacturer's instructions. The endogenous peroxidase activity was removed with 3% H_2_O_2_ for 10 minutes. The samples were then blocked with 10% normal horse serum. Avidin and Biotin blocking solutions were added for 15 minutes, followed by overnight incubation with the primary antibody at 4°C. The secondary antibody was added for 60 minutes, followed by incubation with the ABC reagent for another 60 minutes. NovaRed staining was performed briefly, and the samples were washed with Tris-Buffered Saline (TBS) during each reagent change. All primary antibody information is shown in Table 3.

### Immunoblotting

Nine days post-infection, *in vitro* cell-organoid models were washed three times with DPBS and then treated with RIPA Lysis Buffer (MedChemExpress) supplemented with a protease and phosphatase inhibitor cocktail (MedChemExpress). After homogenization, the samples were centrifuged at 12,000 g for 10 minutes at 4℃. The resulting supernatants were collected for electrophoresis on 10% or 12% SDS-PAGE gels. Then proteins were transferred to a PVDF membrane and blocked with 5% nonfat milk. After incubation with the primary antibody overnight at 4℃, the proteins were incubated with the secondary antibody for 1 hour at room temperature. Protein blots were visualized using an enhanced chemiluminescence assay kit (Thermo Fisher Scientific) and images were captured using a ChemiDOC XRSP System (BioRad). Details of the primary antibodies used are provided in Table 3.

### Detection of cell viability

Cell viability was determined by LIVE/DEAD fluorometric assay (Molecular Probes, Eugene, OR) or the CellTiter-Glo 3D Cell Viability Assay (Promega, G9682) as described by the manufacturer.

After 8 days of infection, living and dead cells were distinguished using fluorescence microscopy according to the procedure provided with the live/dead Viability/Cytotoxicity Kit. Three organoids were randomly selected and the mean fluorescence intensity of labeled living (green) and dead (red) cells was counted. The percentage of live cells was calculated, and mean values ± S.D. of triplicate organoids were obtained.

In order to detect the ATP content of organoids, on the 8th day, the 100µL CellTiter-Glo 3D Cell Viability reagent was added to the above-mentioned lung organoids medium, the medium was shaken to fully mix the reagents, and incubated in a constant temperature incubator for 30 minutes. After completion of the incubation, the lysed organoids were transferred to a 96-well plate compatible with GloMax Promega GloMax Microplate Luminescence Detector, and the microplate luminescence values were determined, and subsequently, the relative viability of the organoids was calculated.

### Statistical analysis

All statistical analyses were performed in “R” and “GraphPad Prism” (GraphPad 7.0) software. The Differentially expressed genes between two groups was assessed by Wilcoxon Rank test. Each *in vitro* experiment was independently repeated at least twice. Data were analyzed as mean ± SEM. Two-sided p values < 0.05 were considered statistically significant.

## Results

### Spatial expression profile of genes in the tissues of pulmonary and intestinal TB

**Figure 1:**
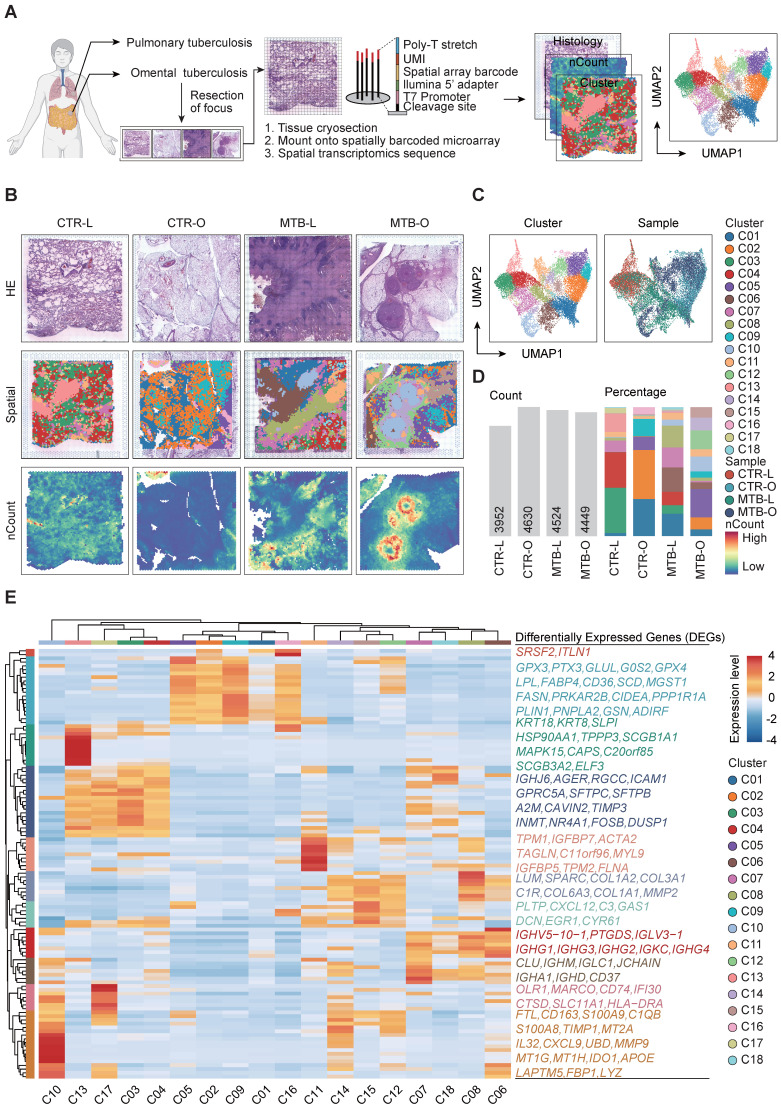
**Comparison of the spatial gene expression of tissuses from TB infected and non-TB infected lung and omentum biopsies. A)** Workflow of the study including sample collection, spatial transcriptomics sequencing and data analysis. **B)** H&E staining, spatial clustering and the nCounts of the 4 tissues. **C)** UMAP plot of all spots from the four tissues and the identified 18 clusters. **D)** Number of Counts in each tissue and the percentage of all cluster in each tissue. **E)** All spots were re-clustered on the basis of genes expression profile.

To comprehensively decode the spatial architecture of the expressed genes in the TB tissues, we performed ST sequencing of the samples from 4 individuals, including one lung (MTB-L) and one omentum TB tissue (MTB-O) along with two non-TB tissues CTR-L and CTR-O as control. Cryosections of the four tissues were mounted on spatially barcoded ST microarray slides and analyzed as illustrated in **Figure 1A**. The distinct pathological features were annotated in adjacent tissue sections after H&E staining by professional pathologists. After clustering and dimensional reduction *via* uniform manifold approximation and projection (UMAP), 18 clusters were identified across the four tissues, in which distinct regions of an enrichment of number of Counts (nCounts) were identified in the tissues of MTB-L and MTB-O (**Figure 1B-C**). nCounts reflect the metabolic proliferation activity of cells in this region. From H&E staining and pathological features, it is recognized that the regions were infiltrated with lymphatic cells that surround the necrotic core. **Figure 1D** shows the detected nCounts and the proportion of each cluster among the four tissues. Thereafter, we re-clustered all spots on the basis of genes expression profile to explore the possible function of each spot. As shown in **Figure 1E**, *GPX3*,* PTX3*,* GLUL*,* G0S2*,* GPX4*,* LPL*,* FABP4*,* CD36*,* SCD*,* MGST1*,* FASN*,* PRKAR2B*,* CIDEA*,* PPP1R1A*,* PLIN1*,* PNPLA2*,* GSN* and* ADIRF* exhibited high expression levels in* C05*,* C02*,* C09*,* C01* and* C16*. As* GPX3* and* GPX4* are recognized as key regulatory factors in the induction of ferroptosis 9 10, the predominant presence in the tissues of CTR-O and MTB-O suggest a potential role of ferroptosis in omentum TB development. *TNXB*, *LTBP4*, *ARGLU1*, *TCF21*, *LPCAT1*, *IGHJ6*, *AGER*, *RGCC*, *ICAM1*, *GPRC5A*, *SFTPC*, *SFTPB*, *A2M*, *CAVIN2*, *TIMP3*, *INMT*, *NR4A1*, *FOSB* and DUSP1 exhibited increased expression in C13, C17, C03 and C04. It is known that *SFTPC* and *SFTPB* are characteristic genes of alveolar epithelium, representing the normal lung tissue component involved in the heterogeneity of tuberculosis granulomas. C07, C18, C08 and C06 exhibited increased expression levels of* IGHV5-10-1*,* PTGDS*,* IGLV3-1*,* IGHG1*,* IGHG3*,* IGHG2*,* IGKC*,* IGHG4*,* CLU*,* IGHM*,* IGLC1*,* JCHAIN*,* IGHA1*,* IGHD* and* CD37*. These proteins belong to the IGH family, which consists of membrane-bound or secreted glycoproteins produced by B lymphocytes 11, 12. C11, C14, C15, C12, C08, and C06 exhibited partial upregulation of *TPM1*,* IGFBP7*,* ACTA2*,* TAGLN*,* C11orf96*,* MYL9*,* IGFBP5*,* TPM2*,* FLNA*,* LUM*,* SPARC*,* COL1A2*,* COL3A1*,* C1R*,* COL6A3*,* COL1A1*,* MMP2*,* PLTP*,* CXCL12*,* C3*,* GAS1*,* DCN*,* EGR1* and *CYR61* genes, respectively. These phenotypes are associated with fibroblast activation and represent the core region of tuberculosis fibrosis. The analysis enabled us to identify the transcriptome characteristics of the spatial distribution during the pathogenesis of primary pulmonary and omental tuberculosis. The accumulation of macrophages and B cells caused by Mtb infection, as well as abnormal activation of fibroblasts, may be important factors in the progression of tuberculosis. This finding led to our focus on the immune cells in the following studies.

### Distinct enrichment pattern of immune cell across different TB-infected tissues

**Figure 2:**
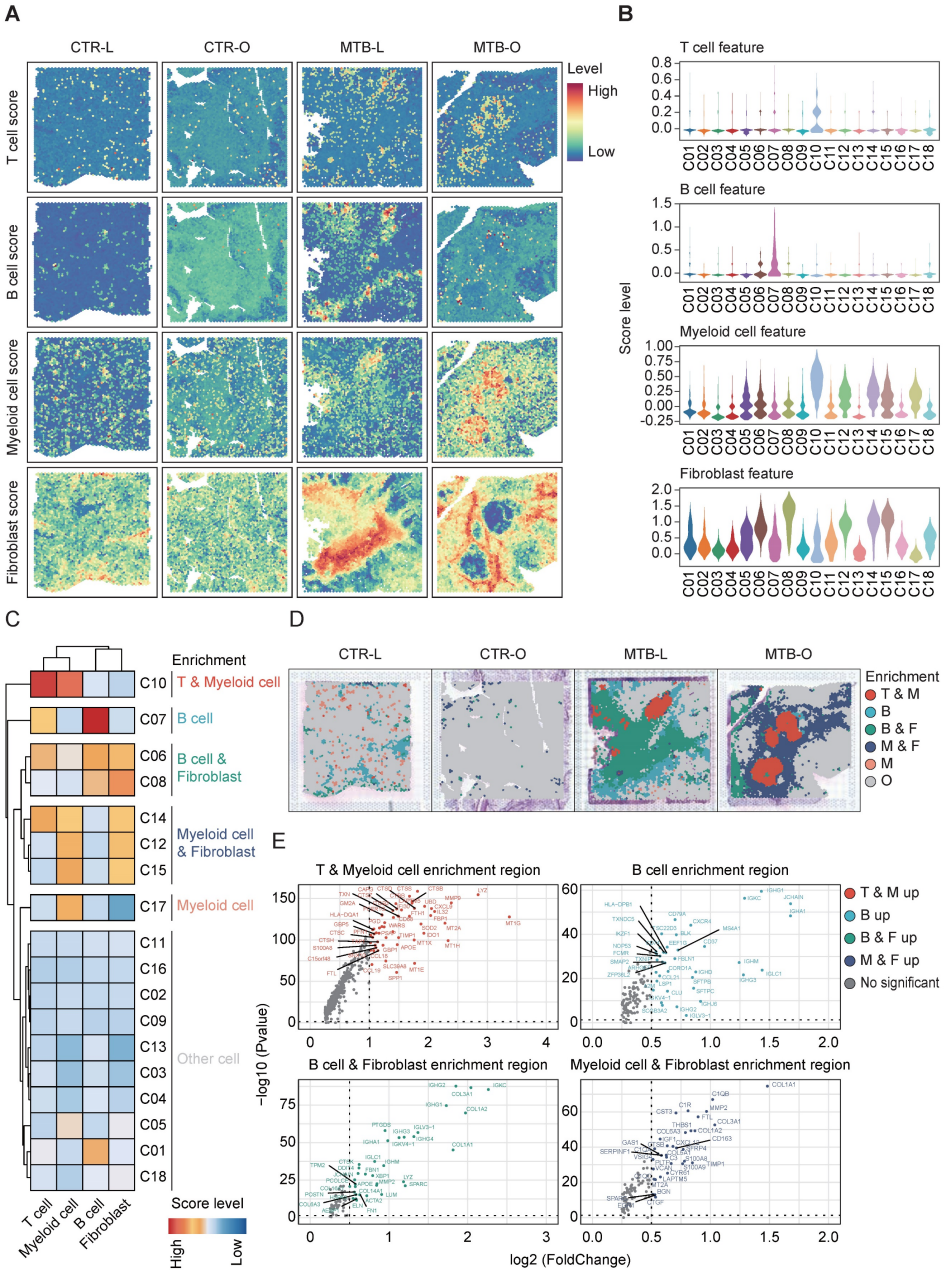
**Distinct enrichment of immune cell across the TB-infected tissues. A and B)** Spatial enrichment analysis of different immune cells showed distinct pattern of immune cell infiltration in the tissues. **C)** The five immune cell enrichment patterns identified after re-clustering all the spots based on the immune cell scoring. **D)** Infiltration of immune cells and the differential immune microenvironment when TB spread from the lung to the peritoneal. **E)** The up-regulated genes in the different enrichment regions of the TB-infected tissues.

Since MTB infection triggers an immune response, leading to the formation of granuloma, understanding of the immune microenvironment in the formation and dissemination of TB remains a topic of significant importance. To address this, we conducted a spatial enrichment analysis that quantified the occurrence of various immune cell subsets. Consequently, the results revealed that T cells and B cells were enriched in C10 and C07, respectively. Myeloid cells were dominant in C10 and fibroblast cells mainly aggregated in C06, C08, C12, C14 and C15 (**Figure 2A-B**). We thereafter re-clustered all the spots and identified five immune cell enrichment patterns based on immune cell scoring. C10 exhibited predominance of both T and myeloid clusters, while C07 showed an exclusive enrichment of B cells. C06 and C08 showed an evident accumulation of both B and myeloid cells. Myeloid and fibroblast cells are simultaneously gathered in C14, C12 and C15. C17 demonstrated a solely elevation of myeloid cells (**Figure 2C**).

By integrating the spatial distribution of the enrichment analysis, the region with T and myeloid cell enrichment was located at the core of TB granuloma found in the MTB-O tissue. The B cell and fibroblast predominant region mostly presented in the MTB-L tissue, however, the myeloid and fibroblast cell enriched locus was dominant among the MTB-O tissue (**Figure 2D**).

This result substantiated a difference of immune microenvironment when TB spread from the lung to the peritoneal. Identification of upregulated differentially expressed genes (DEGs) in different enrichment regions demonstrated that T and myeloid cell enrichment region had a high expression level of *LYZ*, which encodes human lysozyme that have bacteriolytic function and enhanced responsiveness of immunotherapies (**Figure 2E**) 13. In B cell enriched region, B cell-related genes were up-regulated, including *IGHG1*, *IGKC*, *IGH1* and* CXCR4*. Feng et al. reported that B lymphocytes migrate to tuberculous pleural fluid *via* the SDF-1/CXCR4 axis actively respond to antigens specific for Mtb 14, manifesting that B cells were critically involved in the pathogensis of TB. In B and fibroblast cell enrichment region and myeloid and fibroblast cell enrichment region, collagen-related genes were simultaneously upregulated, since fibrous tissue hyperplasia was one of the pathological features of TB 15. The immune score and enrichment analysis of tuberculosis foci revealed the heterogeneity of the immune environment in different locations, specifically the mixed infiltration of macrophages and B cells with fibroblasts in varying proportions in lung tissue and omentum during the progression of tuberculosis.

### Spatial trajectory inference revealed a change of immune microenvironment of TB in lung and omentum

**Figure 3:**
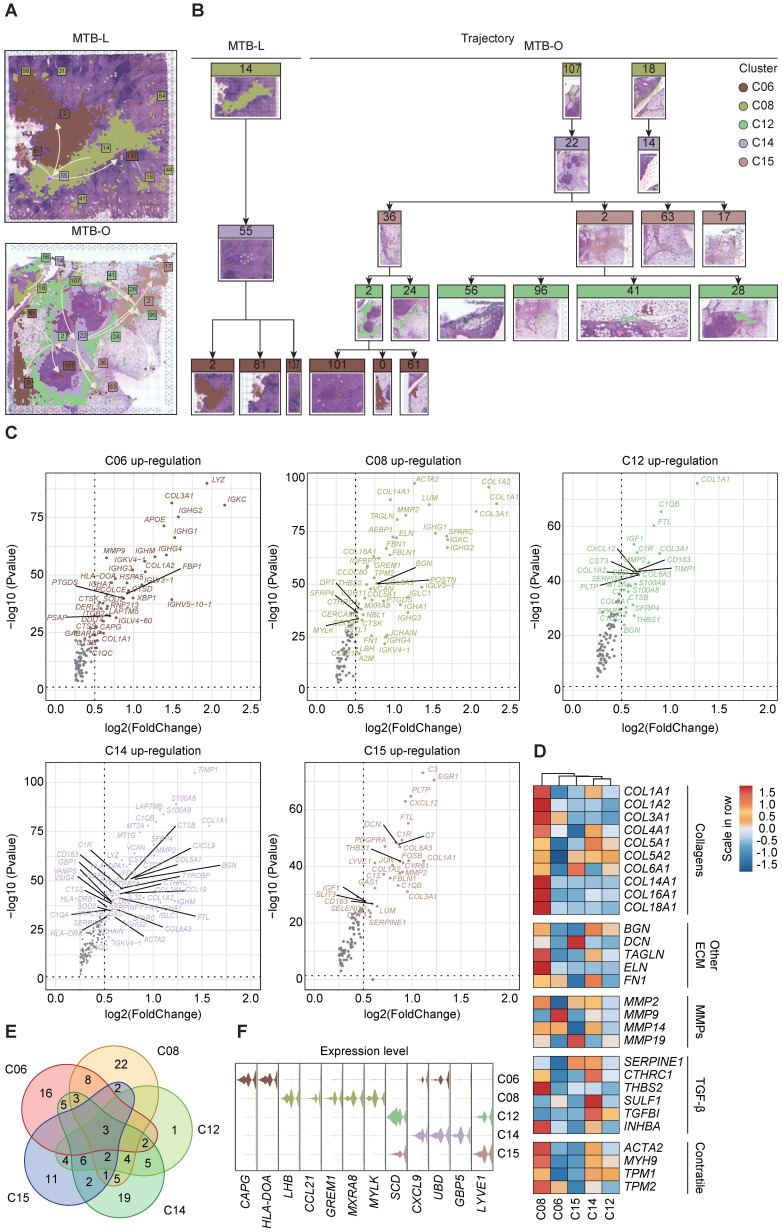
**Spatial trajectory inference analysis of the immune microenvironment of the two TB-infected tissues. A and B)** Pseudo-time spatial trajectory of immune microenvironment revealed a switch from B cell predominance to myeloid cell enrichment in the progression from lung TB to peritoneal TB. **C)** Up-regulated genes were listed from five clusters and collagen-related genes were found to be up-regulated, indicating a fibroblast enrichment feature. **D)** Fibroblast associated genes in the five clusters were demonstrated. **E)** The venn diagram show that 3 genes were all up-regulated in the five clusters. **F)** based on the gene expression pattern, we annotated 5 TB-specific fibroblast clusters into *CAPG^+^* fibroblast (C06), *LHB^+^* fibroblast (C08), *SCD^high^* fibroblast (C12), *CXCL9^+^* fibroblast and *LYVE1^high^* fibroblast.

To clarify the alteration and switch of immune microenvironment from pulmonary TB to peritoneal TB, the pseudo-time spatial trajectory algorithm was applied to map the spatial and transcriptional connections among the clusters (**Figure 3A**). In MTB-L tissue, the spatial trajectory started from C06, then transfer to C14, with final differentiation to C06 (**Figure 3B**). While in MTB-O, the spatial trajectory also started from C06, and a fraction of C06 evolved to C15, followed by transition to C12. A cluster of C12 eventually evolved into C06 (**Figure 3B**). These results indicated a switch from B cell predominance to myeloid cell enrichment in the progression of peritoneal TB. The up-regulated genes of those five clusters were shown in **Figure 3C**. POSTN was also identified in C08. It is a secreted matricellular protein, involves in many fundamental biological events and could induce fibroblasts proliferation 16, 17. Collagen-related genes were found to be up-regulated in C12, C14, and C15, indicating a fibroblast enrichment feature in these clusters. However, MMP2 was also elevated in these clusters 18. Collagen is associated with stability and delimitation of TB granuloma, and an excessive production of MMP2 could render degradation and destruction, causing the release of bacilli and promoting the spread of TB, which partially explain the upregulation of *MMP2* in MTB-O. Next, we demonstrated the fibroblast associated genes in the five clusters (**Figure 3D**) and the venn diagram show that 3 genes were all up-regulated in the five clusters (**Figure 3E**). And based on the gene expression pattern, we annotated 5 TB-specific fibroblast clusters into *CAPG^+^* fibroblast (C06), *LHB^+^* fibroblast (C08), *SCD^high^* fibroblast (C12), *CXCL9^+^* fibroblast and *LYVE1^high^* fibroblast (**Figure 3F**). The analysis of cell development trajectory process has clarified that C12 and C15 are enriched with macrophages and presented in the middle-late stage of nodule formation. The increase in macrophages during the progression process and their substitution for B cells may play a promoting role in the activation of fibroblasts.

### Interaction of THBS1-CD36 between macrophages and fibroblasts

**Figure 4:**
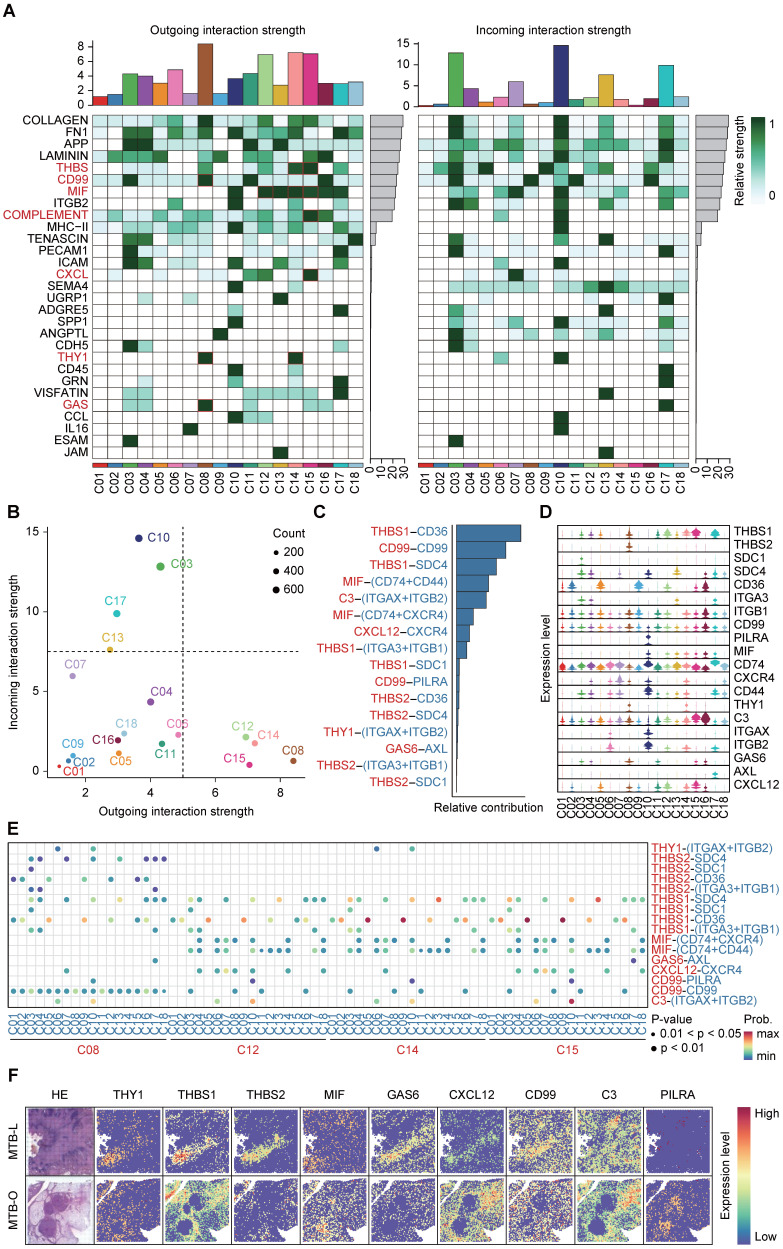
** Interaction of THBS1-CD36 between macrophages and fibroblasts. A)** CellChat analysis of cell-cell communication by mapping incoming and outgoing interactions between all clusters. **B)** Analysis based on interaction strength shows the correlation between incoming and outgoing cells involved in the main signaling pathways in the fibroblast formation in the core area of tuberculosis. **C and D)** The dominant interaction strength in all clusters and the THBS signaling pathway. **E)** THBS1-CD36 interaction was strongest between clusters. **F)** Spatial expression of the critical genes involved in the pathways between cell-cell interaction.

To investigate the precise mechanisms underlying changes in immune microenvironment and fibrosis promotion in TB dissemination, we analyzed cell-cell communication by mapping incoming and outgoing interactions between all clusters (**Figure 4A**). Here, we focus on the main signaling pathways involved in the fibroblast formation in the core area of tuberculosis, such as THBS, CD99, MIF, COMPLENENT, CXCL, THY1, and GAS pathways. We divided the clusters into 4 quadrants based on interaction strength. Clusters C08, C12, C14 and C15 in the lower right corner exhibited the strongest capacity in releasing signals and shaping the microenvironment, while clusters C10, C03, C17 and C13 in the top left corner were mostly 'receiver' subset shaped by outgoing signals (**Figure 4B**). Meanwhile, **Figures 4C** and **4D** presented detailed interaction strength and expression in all clusters. The THBS signaling pathway is widely present in the region enriched with macrophages in the tuberculosis spot, indicating that macrophages and fibroblasts interact depending on THBS1/2-CD36/SDC4/ITGA3/ITGB1, which may be a key pathway for tuberculosis progression. Particularly, the THBS1-CD36 interaction was strongest between C14-C05, C14-C09, C15-C05 and C15-C09 (**Figure 4E**).

Next, we examined critical genes for the spatial expression of each pathway (**Figure 4F**). THBS1 functions as an adhesive glycoprotein mediating cell-to-cell and cell-to-matrix interactions 19, 20. When THBS1 binds to CD36, it can mediate the activity of the TGF pathway and promote fibrosis 21.

Therefore, the progression of tuberculosis fibrosis may not rely on the direct action of Mtb, but rather on the abnormal activation of fibroblasts by recruited macrophages through the THBS signaling pathway.

### THBS1 serves as a target for attenuating fibrosis formation in granulomatous

**Figure 5:**
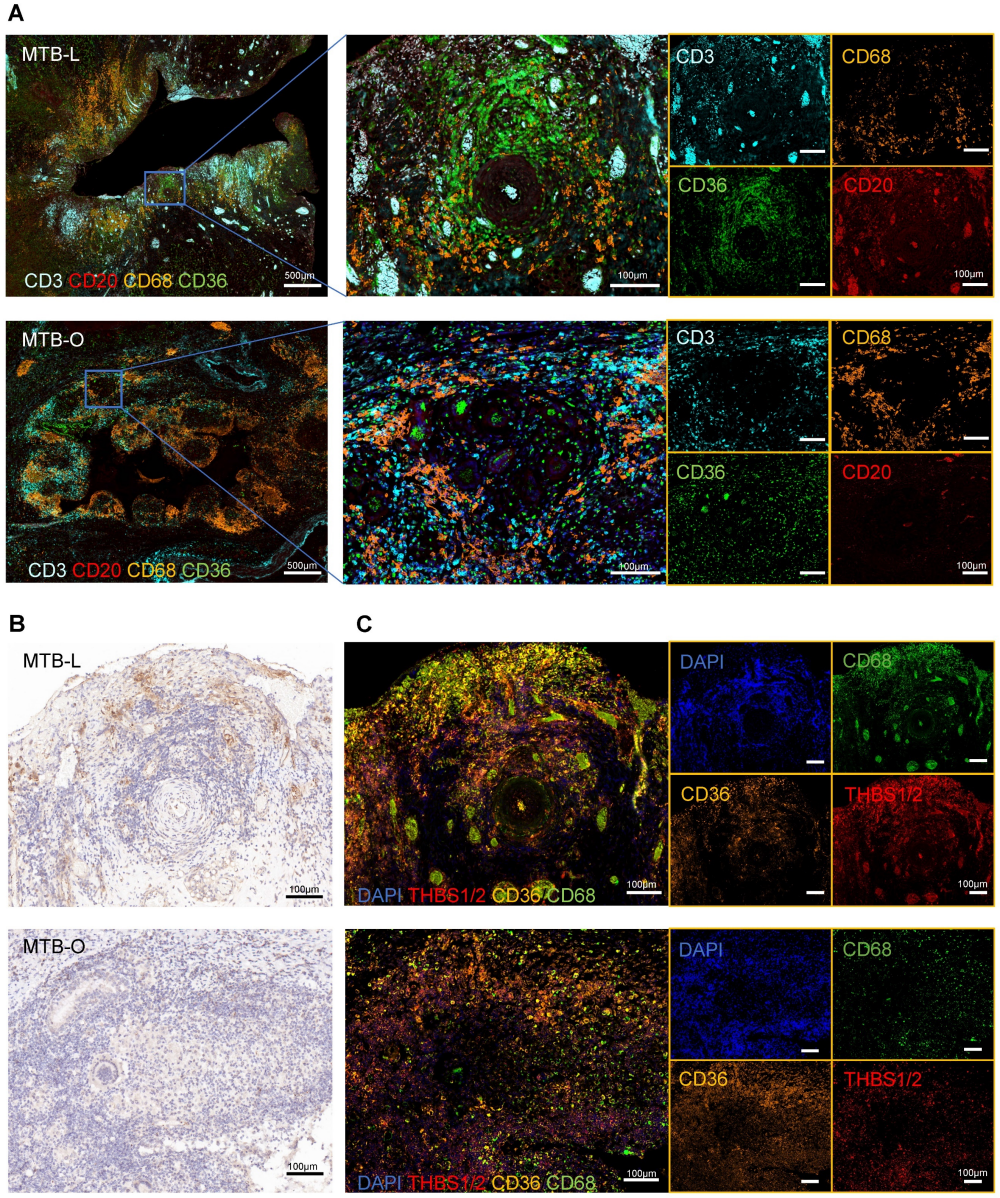
** THBS1 was mainly expressed in macrophages. A)** Multiplex immunofluorescence staining of the TB-infected tissues showed that CD68^+^ macrophages were abundantly clustered in the central region of granulomas, where CD3^+^ T cells and CD20^+^ B cells were infiltrated; in addition, CD36^+^ fibroblasts were also found to predominantly enriched at the core region of the granuloma, forming a ring-like cuff-like structure. Scale bar=500 μm or 100 μm, as indicated in the image. **B)** Immunohistochemical images indicate that THBS1/2 was highly expressed in the granulomatous areas of both lung and omentum tissues. Scale bar=100 μm. **C)** The *in situ* fluorescence hybridization (FISH) showed that THBS1/2 and CD68 were colocalized, indicating that THBS1/2 was mainly expressed in macrophages. Scale bar=100 μm.

Subsequently, we utilized tissue specimens from tuberculosis patients to perform multiplex immunofluorescence, aiming to characterized the compositions of immune cells and fibroblasts in granulomas. The representative images showed that CD68^+^ macrophages were abundantly clustered in the central region of granulomas in both lung and omentum tissues (**Figure 5A**), typically found in the innermost layer of the granulomas 22. CD3^+^ T cells and CD20^+^ B cells also infiltrated the granulomatous area 23. CD36^+^ fibroblasts were also found to be predominantly enriched at the core region of the granulomas, forming a ring-like cuff-like structure (**Figure 5A**). Although fibroblasts are not immune cells, they can also play an important role in the formation of granuloma 24. Once activated, fibroblasts could secrete collagen and cohesion to facilitate the remodeling of the granuloma structure, restraining pathogens in the core region of granuloma to prevent dissemination. Nevertheless, it should be noted that excessive extracellular matrix remodeling can reduce immune cell infiltration, interfering with drug delivery and dampening bacterial clearance efficiency 3, 25. Studies have reported that the interaction between THBS1/2 and CD36 is an important mechanism of TGF-β-mediated fibrosis 26, which has been studied in diseases such as organ fibrosis and tumor extracellular matrix remodeling 27, 28. Our results showed that THBS1/2 was highly expressed in granulomatous areas in both lung and omentum tissues **(Figure 5B)**. Meanwhile, *in situ* fluorescence hybridization (FISH) showed that THBS1/2 and CD68 were colocalized, suggesting that THBS1/2 was mainly expressed in macrophages **(Figure 5C)**.

**Figure 6:**
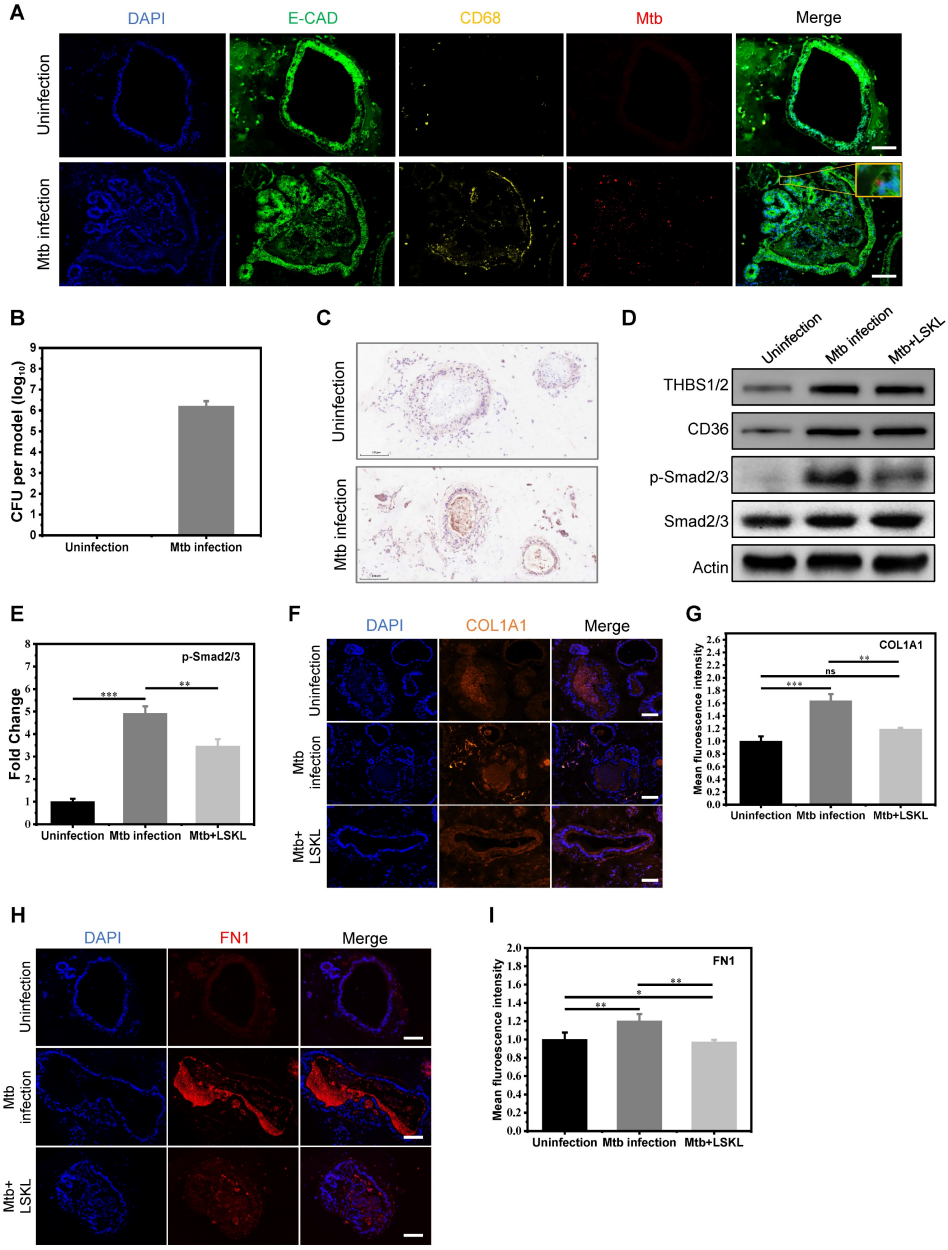
** The THBS-1 inhibitor, LSKL, effectively attenuated fibrosis through inhibition of TGF-beta phosphorylation in Mtb infected organoid models. A)** Mtb infected macrophage-lung organoid co-culture system. E-CAD represents lung organoid, CD68 represents macrophage, and Mtb represents *Mycobacterium tuberculosis*. The yellow square displays the local magnification of the organoid infected with Mtb. Scale bar=100μm. **B)** Quantification of bacterium following Mtb infection in a lung organoid-macrophage model using ImageJ software. n=3. **C)** Immunohistochemical staining of THBS in the lung organoid-macrophage co-culture model with or without Mtb infection. Scale bar=100μm. **D)** Western blot analysis of THBS1/2, CD36 and TGF-β downstream Smad2/3 expression in the control group, Mtb infection group, and Mtb+LSKL treatment group. n=3. **E)** Quantitative analysis of immunoblot bands of phosphorylated Smad2/3 in three groups using ImageJ software. n=3. **F)** Immunofluorescence staining of COL1A1 in the control group, Mtb infection group, and Mtb+LSKL treatment group. Scale bar=100μm. **G)** Quantitative analysis of immunofluorescence of COL1A1 in different groups using ImageJ software. n=3. *P < 0.05, **P < 0.01, ***P < 0.001. **H)** Immunofluorescence staining of FN1 in the control group, Mtb infection group, and Mtb+LSKL treatment group using ImageJ software. Scale bar=100μm. **I)** Quantitative analysis of immunofluorescence of FN1 in different groups. n=3. *P < 0.05, **P < 0.01, ***P < 0.001.

To further explore the possibility of targeting THBS1/2 for the treatment of granulomatous fibrosis, we developed an *in vitro* model of pulmonary tuberculosis by co-culturing macrophages and pulmonary organoids infected with Mtb. The human iPSC-derived lung progenitor cells self-assembled into vacuolar cystic structures with the support of Matrigel, ultimately differentiating into lung organoids. Well-established lung organoids expressed various lung cell type-specific markers, including a variety of epithelial cells and fibroblasts **(Figure S1).** After maturing, the lung organoids were harvested from the Matrigel and co-cultured with macrophages derived from THP-1 cells. The co-cultured system was infected with Mtb and replanted in the Matrigel to construct an *in vitro* model of pulmonary tuberculosis. Immunofluorescence and acid-fast staining revealed the co-localization of Mtb with both fibroblasts and epithelial cells (**Figure 6A and Figure S2A**). Furthermore, the confirmation of Mtb infection in lung organoids model was further validated through the isolation and cultivation of Mtb (**Figure 6B and Figure S2B**). Assessment of cell viability in this model was tested using ATP detection and LIVE/DEAD staining. The results showed that Mtb infection led to a slight decrease in lung organoid viability by approximately 15%; however, overall viability remained relatively high (**Figure S2C-E**). These results indicated that we have successfully constructed an *in vitro* model of pulmonary tuberculosis. Immunohistochemical staining revealed elevated expression of THBS1/2 in the *in vitro* tuberculosis model (**Figure 6C**). The potential of targeting THBS-1/2 for the treatment of fibrosis caused by Mtb was investigated. LSKL, an inhibitor that disrupts THBS1 binding to TGF-β, was employed in the treatment of the M. tuberculosis infection model. Western blot analysis demonstrated increased levels of THBS1/2, CD36, and phosphorylated Smad2/3, with no change in total Smad2/3 protein expression (**Figure 6D-E**). Following LSKL treatment, a significant reduction in phosphorylated Smad2/3 levels was observed (**Figure 6D-E**). These results indicate that Mtb infection can activate TGF-β signaling by inducing the expression of THBS1/2 and CD36. Given the significance of fibronectin 1 (FN1) and collagen in the progression of extracellular matrix (ECM) remodeling within tuberculosis granulomas, our study aimed to assess their expression levels during Mtb infection. Immunofluorescence analysis demonstrated an increase in FN1 and collagen following Mtb infection, with a subsequent decrease observed after treatment with LSKL (**Figure 6F-I**). These findings point to THBS1 as a potential therapeutic target for alleviating granulomatous fibrosis.

## Discussion

Tuberculosis, a contagious human disease caused by Mtb, remains a considerable threat to public health 1. Using clinical samples from two different Mtb-infected organs, we performed spatial transcriptomics to generate a spatial atlas of lung and peritoneal TB tissues, identifying 18 clusters across the two tissue types. Applying the cell enrichment score, we observed B cell enrichment in lung granulomas, while myeloid cells were enriched in omental TB, indicating an immune microenvironment change during peritoneal TB development. The cell-cell communication analysis revealed the leading role of THBS1-CD36. We developed the co-culture model of lung organoids, macrophages and Mtb to further confirm that THBS1 mediates TGF-β signaling to control fibrosis. LSKL, an inhibitor targeting THBS1 binding to TGF-β, was found to reverse the process, implying the clinical application of targeting THBS1 to treat the fibrosis during TB.

Granulomas represent the pivotal pathological structure in tuberculosis with highly variable cell composition 29. In this study, we collected four tissues from two different Mtb infected organs-lung and omentum. The spatial transcriptomics sequencing revealed differences in the immune microenvironment between the two organs. Peritoneal TB is thought to develop from the reactivation of latent foci of infection established in the peritoneum, spreading hematogenously to the mesenteric lymph node from the previous pulmonary infection 6. Our findings revealed an enrichment of B cells in lung granulomas, while myeloid cells were predominantly enriched in omental TB. It has been reported that granulomas were primarily composed of T cells and myeloid cells in most lesions, with subtle variations observed across different organ sites 30. Nevertheless, variations were noted among different TB disease states, suggesting that alterations in the immune microenvironment might originate from the diverse clinical statuses of TB patients.

In our results, fibroblasts and myeloid cells were apparently enriched in the peritoneal TB tissues. Myeloid cells, including macrophages, generally represent the most significant infected host cell and can be triggered to launch innate immune defense against Mtb 31. And fibroblasts could be stimulated by the infected myeloid cells to secret MMPs that will lead to the disruption of basement membranes 32. The presence of both myeloid cells and fibroblasts might indicate the active phase of Mtb infection. On the other hand, our results revealed an enrichment of B cells in the lung granuloma. Growing experimental evidence supports an essential role in immunity against TB. B cells can contribute to the control of TB by producing various cytokines like IL-10, IL-2 and IL-17 33, 34. And B cells are involved in the regulation of neutrophil recruitment to the lungs during Mtb infection 35. B cells can present antigens to T cells and express co-stimulatory molecules that effectively modify T-cell responses 36. We also identified CXCR4 as regulatory factor that mediates B cell function in TB.

Fibroblasts are typically located at the periphery of the granuloma and are ideally poised to direct cell recruitment into the granuloma. However, few studies have investigated the function and mechanism of fibroblasts in TB. In our study, we identified 5 distinct TB-related fibroblast clusters. C06 represented CAPG+ fibroblast. CAPG plays an important role in macrophage function and might have a synergistic effect with myeloid cells 37. CCL21+ fibroblasts have been reported to be involved in T cell activation in lymph nodes during Mtb infection 38. SCD^high^ fibroblasts were first identified in TB tissues and further studies should be carried out to determine their function in TB. CXCL9 has been proved to be associated with TB 39, but the secretion of CXCL9 by fibroblasts had not been reported in TB. LYVE1 is a ligand-specific transporter trafficking between intracellular organelles and the plasma membrane, and was identified as a biomarker for diagnosis of tuberculosis 40. The actual function and mechanism of these fibroblasts remains to be uncovered.

Through cell-cell communication analysis, we identified a dominant interaction between THBS1 and CD36. CD36, a type 2 cell surface scavenger receptor, is widely expressed in many immune and non-immune cells, including macrophages, monocytes, dendritic cells (DCs), T and B cell subsets CD36, also known as platelet glycoprotein IV, mediates THBS and collagen induced cell adhesion, and also participates in the innate immune response to infections 41. The interaction of THBS1-CD36 might have a dominant effect during the fibrotic process associated with tuberculosis. It has been evidenced that the interaction among THBS, CD36 and TGF-β in hepatocytes leads to the activation of TGF-β/Smad signaling pathway, suggesting that inhibiting THBS1 could mitigate collagen fibrosis. To explore the therapeutic potential of targeting THBS1, a co-culture model of human lung organoids and Mtb was developed 42. Lung organoids have greater potential to develop novel diagnostics and therapies for a wide range of diseases 43, such as chronic obstructive pulmonary disease (COPD) 44 modeling influenza virus infections, etc 45. In addition, considering that macrophages play a significant role in TB, we developed a novel lung organoid-macrophage co-culture system and infected it with Mtb to mimic the ECM remodeling process characteristics during Mtb infection, resembling granuloma-associated fibrosis. The *in vitro* culture of lung organoids and immune cells further promotes research on drug screening and disease mechanisms 46, 47. In the current study, we employed this co-culture system to investigate the therapeutic potential of targeting THBS1 in fibrosis treatment. THBS1 inhibitor, LSKL, could significantly reduce ECM remodeling induced by macrophage-fibroblast interaction during TB, suggesting that targeting THBS1 could be a novel approach for TB treatment. Notably, lung organoids exhibit a rich variety of epithelial cell types. In the early stages of tuberculosis infection, epithelial cell, as innate immune cell types, play an important role in promoting cell recruitment and microorganism-killing by releasing innate immune molecules such as SP-A, SP-D, complement C3, antimicrobial peptides, etc 48. Furthermore, proximal airway and distal alveolar epithelial cells in lung tissue participate in regulating the adaptive immune response to mycobacterial infection by producing cytokines such as TNF (tumor necrosis factor), IL-8 (interleukin-8), and GM-CSF (granulocyte macrophage colony-stimulating factor), thereby enhancing cell-cell interaction and activating alveolar macrophages 49. Different macrophage states may play opposite roles in inflammation and tissue repair, such as M2 macrophages releasing TGF-β to facilitate epithelial mesenchymal transition (EMT) and promoting fibrosis progression, as observed in gastric cancer-derived mesenchymal stromal cells inducing M2 macrophage polarization that promotes metastasis and EMT in gastric cancer.

Overall, organoids present a more physiologically relevant model for studying tuberculosis compared to traditional 2D cell cultures, showing promise for translational and personalized medicine. To the best of our knowledge, this is the first study using lung organoid-macrophage co-culture model to characterize Mtb infection. The research successfully profiles the spatial transcriptome of lung and peritoneal tuberculosis, revealing distinct immune microenvironmental changes between these sites. Additionally, we discovered a potential therapeutic target, THBS1, which, combined with conventional anti-TB chemotherapy, may be a promising adjuvant therapy for alleviating fibrosis outcomes and improving organ function in TB patients. Furthermore, THBS1 has been shown to enhance the susceptibility of Gram-positive bacteria to infection by other researchers. Therefore, it is necessary to further comprehensively evaluate this treatment regimen, particularly focusing on its impact on inflammation, bacterial infection, and *in vivo* replication, to fully understand the benefits of this treatment strategy.

The study's limitations included a small sample size and insufficient validation of findings *in vivo*. The resolution of spatial transcriptome sequencing still involves a mixture of several cells, which cannot achieve true single-cell level precision. The surrounding cells of omental tuberculosis mainly consist of adipose vacuolar cells, lacking spatial structural interaction with adjacent tissue cells.

## Conclusion

Using spatial transcriptomics, we examined the cell composition and spatial distribution characteristics of tuberculosis granulomas in lung and omental tissues, accurately summarized the characteristics and evolutionary relationships of major cell populations in granulomas, defined key differential genes in cell clusters in different regions, and found that up-regulation of collagen gene is a common feature of granulomas. In addition, we demonstrated the positive effect of THBS1 intervention by using the established *in vitro* TB model based on organoids and macrophages co-culture, providing new ideas for future TB treatment.

## Supplementary Material

Supplementary figures.

## Figures and Tables

**Table 1:** Clinical tuberculosis samples information

Sample ID	Group	Age	Sex	AFB	PCR	HIV
255109	MTB-L	37	W	Positive	Positive	Negtive
247331	MTB-O	18	W	Positive	Positive	Negtive
266999	CTR-L	46	W	Negtive	Negtive	Negtive
273044	CTR-O	32	M	Negtive	Negtive	Negtive

**Table 2:** Probe sequence information for fluorescence *in situ* hybridization.

Target name	Probe sequence (5*′-*3*′*)	Fluorescence Channel
CD36	CAACTGGCATTAGAATACCTCCAAACAC, GTAGTAACAGGGTACGGAACCAAACTCA, CCTTTATATGTGTCGATTATGGCAACTTTAC, AGATTAACGTCGGATTCAAATACAGCATA, AATGGTCCCAGTCTCATTAAGCCAAAG	Cy3
THBS1/2	GTGAGTTCAAAGATGTCAAACACGCTG, CCTGAGGATGTCTTCTGGTGTGGTTC, AGTGACACTCAGTGCAGCTATCAACAGT, ATCAGGGTAGCTAGTACACTTCACGCC, GCCAGCGTAGGTTTGGTCATAGATAGG, TGATACTGAAAAGGTCGAAGGTCGTGT, GAAGCAAACCCCTGAAGTGACTCTCTC, GAAACTGGTTATCATTCGACACTCTCTTG	Cy5
CD68	TGTCCATAGGGGAATGAGAGAAGCAG, GTGTCCATAGGGGAATGAGAGAAGCA, GATGATGAGAGGCAGCAAGATGGAC, TTTTGTTGGGGTTCAGTACAGAGATGC	FITC

**Table 3:** The primary antibodies used in this study.

Primary antibodies	Source	Cat#	Application
SFTPC	Santa Cruz	sc-518209	Immunofluorescence
E-cadherin (E-CAD)	BD Biosciences	610181	Immunofluorescence
FOXJ1	Invitrogen	14-9965-82	Immunofluorescence
MUC5AC	Abcam	ab198294	Immunofluorescence
P63	Abcam	ab124762	Immunofluorescence
SCGB3A2	Abcam	ab181853	Immunofluorescence
Podoplanin (PODN)	Abcam	ab10288	Immunofluorescence
THBS1/2	Santa Cruz	sc-133061	Immunohistochemistry/ Immunofluorescence/Western Blot
CD36	Abcam	ab133625	Immunohistochemistry /Immunofluorescence/ Western Blot
CD68	BioLegend	333802	Immunofluorescence
CD3	Abcam	ab16669	Immunofluorescence
CD20	Abcam	ab78237	Immunofluorescence
Fibronectin1 (FN1)	Santa Cruz	sc-8422	Immunofluorescence
Mtb	BioRad	OBT0947	Immunofluorescence
COL1A1	Abcam	ab138492	Immunofluorescence
p-Smad2/3	Abcam	ab272332	Western Blot
Smad2	Abcam	ab217553	Western Blot
Actin	CST	8H10D10	Western Blot
