# Supplementary material for: Spatial transcriptomic sequencing reveals immune microenvironment features of Mycobacterium tuberculosis granulomas in lung and omentum
Source: Theranostics. 2024 Sep 23;14(16):6185–201. doi: 10.7150/thno.99038 (PMC11488093; doi:10.7150/thno.99038)
Supplement: Supplementary file 1 — Supplementary figures. [file thnov14p6185s1.pdf]

Figure S1. Establishment of human iPSCs derived lung organoids

A) Schematic diagram of the pulmonary organoid differentiation protocol. iPSC: induced pluripotent stem cells; DE: endoderm; AFE: foregut endoderm; LPC: lung tissue cells; 3D: lung 3D spheroids; Branching: lung branching stage; Mature: lung maturation stage.

B) Representative brightfield image of mature lung organoid. Scale bar=100 μm.

C) Immunofluorescence identification reveals multiple lung cell types in lung organoids. DAPI: nucleus; SFTPC: type II alveolar functional protein markers, alveolar surfactant protein C; PODN: type I alveolar cell marker; MUC5AC: goblet cells; a-SMA: fibroblasts; Vimentin: mesenchymal cells; SCGB3A2: club secretory cells; FOXJ1: ciliated cells; E-CAD: epithelial cells; P63: basal cells. Scale bar=20 μm.

Figure S2. Establishment of an effective *in vitro* model for pulmonary tuberculosis

A) Acid fast staining for identification of Mtb in infected lung organoids. Scale bar=50 μm.

B) Mtb cultures derived from the pulmonary tuberculosis *in vitro* model grown on agar slant medium.

(C) Representative images of LIVE/DEAD staining pre- and 8 days post-infection of human lung organoids. Scale bar=250 μm.

D) Fluorescence intensity analysis of LIVE/DEAD staining. n=3.

E) Analysis of ATP luminescence intensity measurements in lung organoids infected with Mtb. n=3.

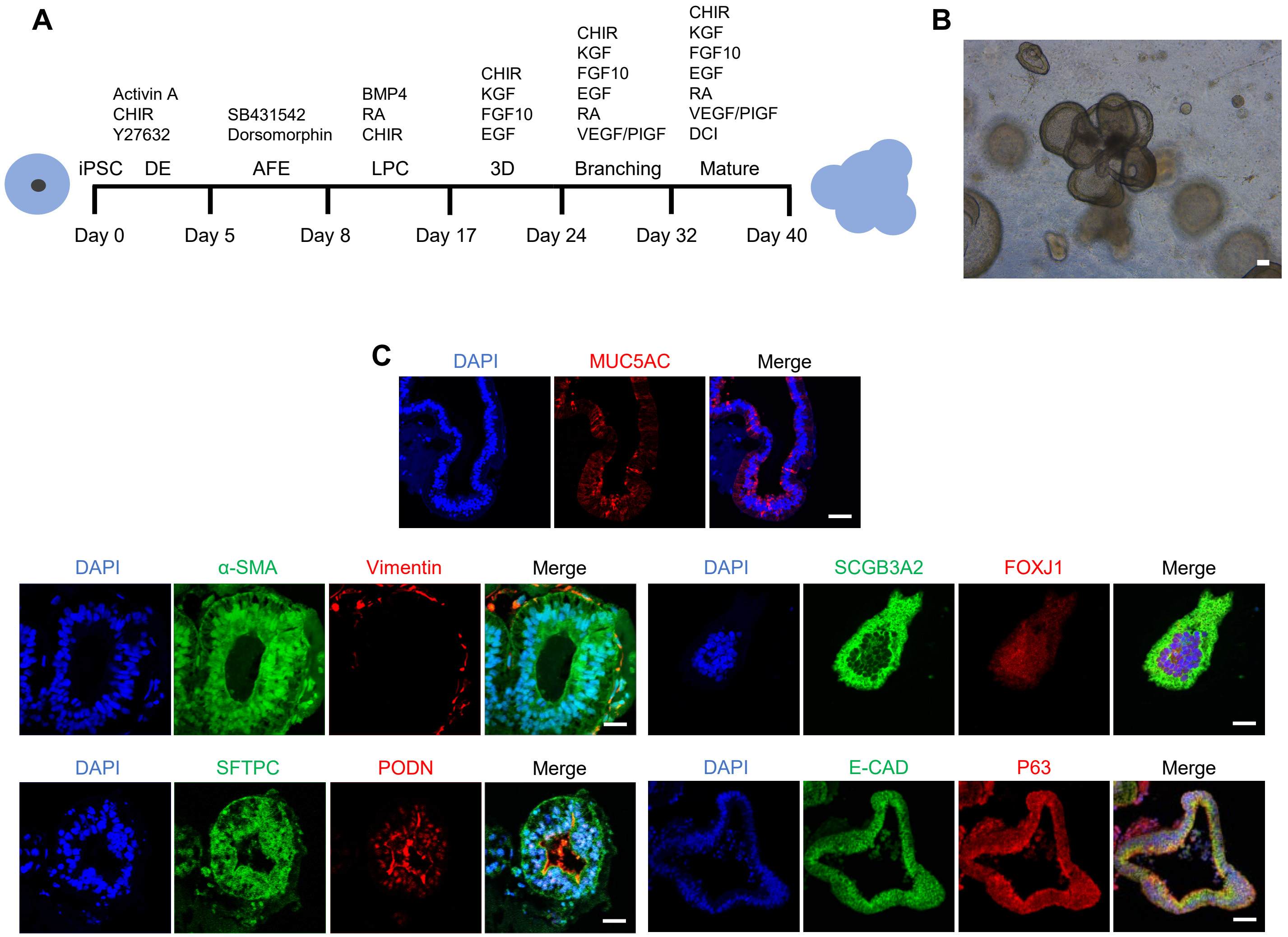
A
Activin A
CHIR
Y27632
SB431542
Dorsomorphin
BMP4
RA
CHIR
CHIR
KGF
FGF10
EGF
CHIR
KGF
FGF10
EGF
RA
VEGF/PIGF
CHIR
KGF
FGF10
EGF
RA
VEGF/PIGF
DCI
iPSC
DE
AFE
LPC
3D
Branching
Mature
Day 0
Day 5
Day 8
Day 17
Day 24
Day 32
Day 40
B
C
DAPI
MUC5AC
Merge
DAPI
α-SMA
Vimentin
Merge
DAPI
SCGB3A2
FOXJ1
Merge
DAPI
SFTPC
PODN
Merge
DAPI
E-CAD
P63
Merge

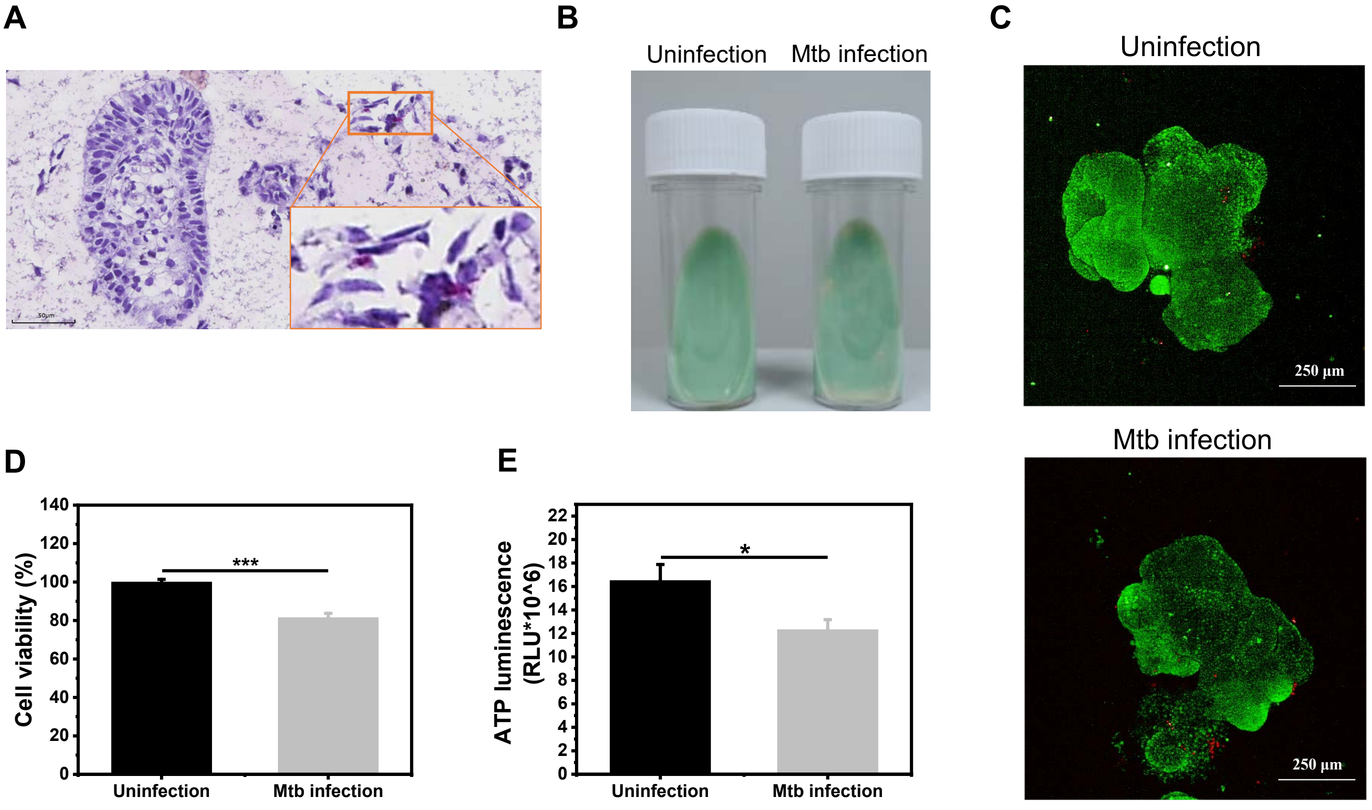
A
B
Uninfection
Mtb infection
C
Uninfection
250 µm
Mtb infection
250 µm
D
Cell viability (%)
140
120
100
80
60
40
20
0
***
Uninfection
Mtb infection
E
ATP luminescence
(RLU*10^6)
22
20
18
16
14
12
10
8
6
4
2
0
*
Uninfection
Mtb infection
